# 

**Published:** 1896-09-19

**Authors:** 


**Notices of Births, Deaths, and Marriages** are inserted in this column at a charge of 2s. 6d. for an announcement not exceeding four lines.

### NOTICE TO CORRESPONDENTS.

All MS., Letters, Books for Review, and other matters intended for the Editor should be addressed The Editor, The Lodge, Porchester Square, London, W.

The Editor cannot undertake to return rejected MS., even when accompanied by stamped directed envelope.

**Notice to Advertisers and Subscribers.**

All Advertisements, Orders for Copies of The Hospital and Business Communications should be addressed to **THE MANAGER, (Not to The Editor)**, The Hospital, 428, Strand, London, W.C.

The Cost of Subscription, post free, is as follows:—Per week, 2½d.; per quarter, 2s. 8d.; per half-year, 5s. 3d.; per annum, 10s. 6d. Foreign:—Per Annum, 15s. 2d.; payable, in all cases, in advance.

Price 5s.

**"Burdett's Hospitals and Charities, 1896."**

Seventh year of publication. Over 1,000 pp. Scarlet cloth, gilt lettered. Price 5s. A most complete guide to British, Colonial, Indian, and American Hospitals, Dispensaries, Nursing and Convalescent Institutions, and Asylums. A few complete sets of the preceding six issues of the "Annual" may still be obtained on application to the Publishers, The Scientific Press (Limited), 428, Strand, London, W.C.

### BETHLEM HOSPITAL.

### SCRAPS AND GLEANINGS.

The Dudley cycle and trades' parade resulted in the raising of £120 for the local charities.

At Retford, on Hospital Sunday, £67 was collected in aid of the Retford Cottage Hospital.

There are only ten houses licensed as retreats for inebriates under the Inebriates Acts of 1879 and 1888.

The amount produced by the street collection in Scarborough on Hospital Saturday there was £270.

There were 371 applicants for the Edinburgh Trinity Hospital Pensions, and there were 18 vacancies.

At the commencement of September the Newcastle New Infirmary Fund had increased to upwards of £12,000.

Over a ton of flowers are said to have been sold on the occasion of Hospital Saturday, in Douglas, Isle of Man.

The first Hospital Sunday parade of the friendly and temperance societies of the town was held at Redhill on the 30th ult.

The system of purchasing provisions by tender has recently been adopted at the General and Marine Hospital, St. Catherine's.

During the past two years nearly 10 per cent. of the total population have been treated each year at the Assam dispensaries as either in or out-patients.

Plans have been prepared for the erection of a new building for the department of dentistry at the University of Pennsylvania. It will cost 120,000 dols.

The new operating-room to be built at the Cumberland Infirmary is to take the form of an extension of the ground floor of the western wing, and will cost about £1,000.

The Tamworth Hospital Saturday street collections in aid of the funds of the Tamworth Cottage Hospital amounted this year to £34 17s., a slight advance upon last year.

The expenditure at the Sheffield General Infirmary for the last year was £808 8s. 8d. in excess of the income, and increased subscriptions are appealed for to wipe off this deficiency.

The returns, as a whole, from the Eastbourne Hospital Sunday collections show a falling-off as compared with last year. The special parade collection was, however, a great success.

The two founders of the Royal Victoria Hospital in Montreal (Lord Mount-Stephen and Sir Donald A. Smith) have given an additional sum of 800,000 dols. towards its permanent endowment.

At the Corbett Hospital, during the past year, 246 patients were treated. The three main items of income, viz., workpeoples' contributions, annual subscriptions, and donations, show an increase.

During the past decade the number of dispensaries in Assam has risen from 46 to 97; while, in the same time, the patients treated has increased from 149,051 to 544,938, or by more than 265 per cent.

The friendly societies' demonstration, on behalf of the North Devon Infirmary, Barnstaple, has this year been a greater success than ever. About £200 has been received, against a previous record of £172 in 1894.

The amount collected in Ilfracombe on Hospital Saturday in aid of the funds of the Ilfracombe Tyrrel Cottage Hospital was, at the end of August, £189 15s. 8½d., but it is expected that the total amount will be over £200.

The principal work of the managers of the Corbett Hospital, at Stourbridge, has been the carrying out of the scheme for providing a children's ward. A six-bedded ward has been built, and arrangements made to set free one of the other wards for children.

The managers of the Newcastle Royal Infirmary have withdrawn their application for the use of the recreation ground for the purposes of their forthcoming exhibition, owing to the report of the Town Moor Management Committee, to which we referred last week.

Surgical cases have during the year 1895 largely predominated at the Wilkes-Barre City Hospital. Eight hundred and eleven patients altogether were treated, of whom 76 per cent. were admitted from injuries. The average stay of a patient in the hospital was 25 days.

From the account of a Japanese army surgeon who has recently visited the Spanish military hospitals in Havana, it would appear that the nurses are merely labourers picked up on the field, "very dirty, and wholly ignorant of the first principles of care for the wounded."

The number of cases treated during the year 1895 at the Jewish Convalescent Homes, at West Norwood, and at Brighton, show an increase when compared with those admitted during 1894. The figures are: West Norwood, 1895, 355; 1894, 314; Brighton, 1895, 229; 1894, 218.

Out of 5,987 in-patients treated in 1895 at the Assam Dispenaries 4,382, or about 73 per cent., were dieted at the cost of the dispensary funds. Of the remainder the dieting of 811, or rather more than one half, was paid for by the managers of tea gardens, of 746 by the patients themselves, and of 48 (viz. police cases) by the Government.

There are five Roman Catholic and one Protestant reformatories, and sixty Roman Catholic and ten Protestant industrial schools in Ireland at the present time. The total expenditure during 1895 on reformatory schools in Ireland was £15,075. and on industrial schools £165,812. The cost per head averaged £26 7s. 7½d. in the reformatories, and £18 10s. 7d. in the industrial schools.

The new buildings at the Rochdale Infirmary are expected to be opened ready for use at the latter end of October. At the monthly meeting of the Infirmary Workmen's Committee held recently, it was announced that there was a balance of £60 to the credit of the committee in the bank, the £250 voted at the last meeting having been paid to the general fund of the Infirmary.

The accounts presented at the annual meeting of the North Lonsdale Hospital showed a very satisfactory state of affairs. The balance in hand this year was over £386 in excess of that of last year. The workmen's ordinary contributions have been increased this year by the sum of £153 14s. 4d. 272 in-patients and 1,604 out-patients have been treated during the past year, making the largest total ever recorded in the books of this institution.

At the enquiry held at the Martley Workhouse recently, into the necessity of an isolation hospital being established for the use of the inhabitants of the district under the Martley Rural Council, a number of communications were received from parish councils and parish meetings, and petitions from ratepayers of various parishes, uniformly disapproving of the proposal. Alderman James Essex was the only one present who spoke in favour of the hospital, which he thought was urgently needed. The report will be laid before the County Council in due course.

### The Student of Medicine.

### APPOINTMENTS.

S. H. B. Allison, B.A., M.B., C.M.Edin., appointed Medical Officer and Medical Officer of Health to Claudy Dispensary District, Londonderry Union. S. H. Harrison, M.R.C.S., L.R.C.P., appointed Medical Officer for the North Walsham District of the Smallburgh Union. H. T. Heath, L.R.C.P.Edin., L.R.C.S.I., appointed Medical Officer of Health to the Mansfield Woodhouse (Notts) District Council. Dr. Hughes (appointed Medical Officer to the Mainday Colliery of the Ocean Company. J.H. Hunt, M.B.Lond., appointed Assistant House-Surgeon to the Halifax Infirmary. J. A. Hutton, M.D., B.S., M.R.C.S., L.R.C.P., D.P.H. appointed Surgeon to the Scarborough Borough Police Force. J. A. Lindsay, M.A., M.D., R.U.I., M.R.C.P.Lond., appointed Physician to the Belfast Royal Hospital. J. H. Ray, M.B., Ch.M.Vict., F.R.C.S.Eng., L.R.C.P.Lond., Senior House-Surgeon to the Salford Royal Hospital, appointed Resident Surgical Officer to the Manchester Royal Infirmary. G. C. Sandford, M.D.Edin., appointed Resident Medical Officer to the Dispensary, York. E. G. B. Starkie, M.B., C.M.Edin., appointed Medical Officer of Health to the Fylde Rural District. J. W. Stokes, M.R.C.S.Eng., L.R.C.P.Lond., appointed House Surgeon to the Children's Hospital, the Wicker, Sheffield. J. Watson, M.D., F.R.C.S.E., D.P.H., &c., appointed Surgeon to the Newbury District Hospital. A. M. Watts, M.R.C.S., L.R.C.P., appointed House-Surgeon to the Hereford General Infirmary. T. P. Grosart Wells, L.R.C.P.Edin., &c., appointed Medical Officer of St. Albans Union Workhouse and No. 1 District.

### VACANCIES.

The following vacancies are announced this week:—

### Resident Medical Officers.

Bethlem Hospital.—Two Resident Clinical Assistants. Term of residence six months from November 1st. Apartments, board, and washing provided. Applications to John Brewer, Clerk, &c., Bridewell Hospital, New Bridge Street, E.C., before October 5th, endorsed "Clinical Assistantship."

Blackburn and East Lancashire Infirmary.—Senior House-Surgeon. Salary, £100 per annum, with board, washing, lodging, &c. Applications to Nathan A. Smith, Secretary, Infirmary Office, 15, Richmond Terrace, Blackburn, by September 24th.

Cheltenham General Hospital.—House Surgeon; unmarried. Must possess a registered qualification in medicine and surgery. Salary £80 per annum, with board and apartments. Also Junior House-Surgeon; must be unmarried and have a registered qualification in Medicine and Surgery. Appointment tenable for two years. Salary, £40 per annum, with board and apartments. Applications to H. T. Carrington, Honorary Secretary and Treasurer, by September 19th for the former and September 26th for the latter vacancy.

Fulham Union.—Assistant Medical Superintendent of the Union Infirmary; must be unmarried and possess a Medical and Surgical qualification. Salary £120 per annum, increasing £10 yearly to a maximum of £150, with board, furnished apartments, attendance, and washing. Applications to T. Aplin Marsh, Clerk to the Guardians, Union Offices, Fulham Palace Road, Hammersmith, W., by September 28th.

General Hospital, Birmingham.—Assistant House-Surgeon; must possess Surgical qualification and be registered. Appointment for six months. No salary, but board and washing provided. Applications to Howard J. Collins House Governor, by September 26th.

Hants County Asylum.—Assistant Medical Officer; unmarried; doubly qualified and registered under the medical Act. Candidates should not exceed the age of 25 years. Salary £100 per annum, increasing, to £125 after twelve months' service, with apartments, board washing, and attendance. Applications forwarded by September 21st, addressed to the Committee of Visitors, Knowle, Fareham, endorsed "Application for appointment of Medical Officer."

Hospital for Consumption and Diseases of the Chest, Brompton.—Resident House-Physician. Applications to W. H. Theobald at the Hospital by September 23rd.

Royal Free Hospital, Gray's Inn Road, W.C.—Two House Physicians. Appointments for six months. No salary, but board, &c., provided. Applications to the Secretary by September 21st.

Somerset and Bath Lunatic Asylums (Western Joint Asylum), Cotford, near Taunton.—Medical Superintendent. Salary £450 per annum, with partly furnished house, coals, light, washing, milk, and garden produce. Applications to John Coates, Clerk to the Visiting Committee, Somerset and Bath Asylum, Wells, Somerset, by October 1st.

### Non-Resident Medical Officers.

General Hospital, Birmingham.—Two Anæsthetists; must possess Medical and Surgical qualifications and be registered. Each appointment for one year, eligible for re-election. Salary, £40 per annum.

Owen's College, Manchester.—Senior and Junior Demonstrator in Physiology. Salary £150 per annum rising to £200, and £100 rising to £150 respectively. Applications to S. Chaffers, Registrar, by September 22nd.

